# Supplementary material for: Compensation or Displacement of Physical Activity in Children and Adolescents: A Systematic Review of Empirical Studies
Source: Children (Basel). 2022 Mar 3;9(3):351. doi: 10.3390/children9030351 (PMC8947494; doi:10.3390/children9030351)
Supplement: Supplementary file 1 [file children-09-00351-s001.zip › Table S1 Data extraction of included studies.pdf]

Table S1 Data extraction of included studies

Author Study	***Study design***	***ParticipaNts***	***INT 1 INT 2 INT 3 CON***	***Purpose of the study***	***Setting***	***Intervention***			***Measurement Instrument Duration***	***measurement periods for compensation***	***indicators for compensation***		
						Stimulus	*Duration*	*Dose*					
**Abuerto et al.; 2011; Mexico**	Cluster RCT	N = 699; 10.2 years; m = 339; f = 360	240	effects of a school based environmental intervention on PA	intervention	two additional PE classes/week<br><br>one additional recess (teacher promotion of PA)	6 months	50min/day<br>2 times/week<br><br>15min/day<br>5 times/week	Step counts<br><br>Pedometer<br><br>5 days	During school<br>Out of school<br>All day	p ≤ 0.05 ↑ during school<br>overall PA ↔<br>supporting compensation	higher during school PA is compensated by less PA outside of school<br>overall PA maintained stabled	INT1:<br>during school ↑*(p≤ 0.05)<br>out of school ↓<br>all day ↔
			242			one additional PE class/week<br><br>one additional recess (teacher promotion of PA)		50 min/day<br>1 time/week<br><br>15min/day<br>5 times/week					INT 2:<br>during school ↑<br>out of school ↔<br>all day ↑
			n.g.										
			217			no change							CON:<br>during school ↓<br>out of school ↑<br>all day ↓

**Adamo et al.; 2017; Canada**<br><br>ABC trial	Cluster RCT	N = 153; 3.6 years; m = 117; f = 36	39	efficacy of an intervention program delivered in licensed childcare (CC) settings with or without the addition of parent-facilitated home PA promotion to increase preschoolers overall PA level	intervent ion	CC+home: ABC trial in childcare center and at home	6 months		MVPA<br>SED<br><br>Accelerometer<br><br>5 days	Total hours<br>CC hours<br>Outside CC hours	p ≤ 0.05 ↑ total PA<br>not supporting compensation	overall PA increased	INT 1:<br>Total PA CC hours ↑<br>Total PA outside CC hours ↑<br><br>MVPA CC hours ↑<br>MVPA outside CC hours ↑* (p ≤ 0.01)<br><br>SED CC hours ↓<br>outside CC hours ↓	
			72			CC: ABC trial only in childcare Center						overall PA increased	INT 2:<br>Total PA CC hours ↑<br>Total PA outside CC hours ↑* (p ≤ 0.05)<br><br>MVPA CC hours ↑* (p ≤ 0.05)<br>MVPA outside CC hours ↑* (p ≤ 0.01)<br><br>SED CC hours ↓<br>outside CC hours ↓* (p < 005)	
			n.g.											

			42			no Intervention						overall PA increased	CON:<br>Total PA CC hours ↑* (p≤0.01)<br>Total PA outside CC hours ↑<br><br>MVPA CC hours ↑* (p≤ 0.05)<br>MVPA outside CC hours ↑<br><br>SED CC hours ↓* (p≤ 0.01)<br>outside CC hours ↓
**Adkins et al.; 2013; USA**	Cluster RCT	N = 232 (all m); 8.1 years; ;	88	Comparison of a before-school group dance video intevention to a running club on changes in SED and MVPA time	intervent ion	Run: before school running on a 400m track	7 weeks	30 min/day 2 times a week	MVPA<br>SED<br><br>Accelerometer<br><br>7 days	morning<br>rest of the day	p ≤ 0.05 supporting compensation	higher morning PA is compensated by higher SED throughout the rest of the day	morning MVPA ↑* (p≤ 0.05)<br><br>rest of the day SED ↑* (p≤ 0.05)
			46			DDR: video game dance intervention	7 weeks						
			53			JD: video game dance intervention	7 weeks						
			45			no Intervention							

**Alderman et al.; 2012; USA**	Cross Sectional Study	N = 279; 11.8 years; m = 159; f = 120		compensatory or stimulatory effects of PE on daily PA	Physical Education				Steps per day<br><br>Accelerometer<br><br>5 days	school day<br>after school	$p \leq 0.05$, ES: large not supporting compensation<br><br>partial:<br>$p \leq 0.05$ supporting compensation in girls<br>total PA on PE day boys = girls	overall PA on PE day higher than on non PE day<br><br>lower PA during PE is compensated by more activity outside of PE	PE day >* non PE day ($p \leq 0.05$)<br><br>total day<br>boys = girls<br><br>PE time<br>boys >* girls ($p \leq 0.01$)
**Alhassan et al.; 2007; USA**	RCT	N = 32; 3.6 years; m = 20; f = 12	17	effects of increasing recess time on total PA levels attending full-day preschool<br><br>compensatory effects of increased reces time during school on after school PA	intervention	increasing recess time	3 months	30 min/day<br>2 times a week	CPM<br>MVPA<br><br>Accelerometer<br><br>4 days	total day<br>during school day	INT = CON supporting compensation	INT group and CON group did not show different PA levels	total daily CPM<br>INT = CON<br><br>during school day CPM<br>INT = CON<br><br>total daily MVPA<br>INT = CON<br><br>during school day MVPA<br>INT = CON<br><br>no sign. changes von Pre to post in INT
			n.g.										

			n.g.										
			15			no intervention							
**Alhassan et al.; 2012; USA**	RCT	N = 71; 4.3 years; m = 35; f = 36	42	effects of classroom teacher taught LMS-based PA program on PA levels	intervent ion	teacher taught LMS based PA	6 months	30 min/day 5 times a week	MVPA<br>LPA<br>SED<br><br>Accelerometer<br><br>7 days	preschool time<br>total day	p ≤ 0.05, ES: medium/small not supporting compensation	overall PA increased	INT 1:<br>during Preschool SED ↓* (p≤ 0.02)<br>during Preschool LPA ↑<br>during preschool MVPA ↑<br><br>total day SED ↓* (p≤ 0.01)<br>total day LPA ↑<br>total day MVPA ↑
			n.g.										
			n.g.										
			28			no intervention							CON:<br>during Preschool SED ↑<br>during Preschool LPA ↓<br>during preschool MVPA ↓<br><br>total day SED ↑<br>total day LPA ↑<br>total day MVPA ↓

**Alhassan et al.; 2013; USA**	RCT	N = 67; 4.1 years; m = 38; f = 29	38	effects of increasing structured outdoor playtime on PA	intervent ion	structured outdoor playtime	4 weeks	30min/day 3 times a week	MVPA<br><br>Accelerometer<br><br>5 days	total day preschool time after preschool	$p \leq 0.05$ ↑ total day not supporting compensation	overall PA increased	INT 1:<br>total day SED ↓* ($p \leq 0.01$)<br>total day LPA ↑<br>total day MVPA ↑* ($p \leq 0.05$)<br><br>during school SED ↓* ($p \leq 0.01$)<br>during school LPA ↑<br>during school MVPA ↑<br><br>afterschool SED ↓<br>afterschool LPA ↑<br>afterschool MVPA ↑
			n.g.										
			n.g.										
			29			no intervention							CON:<br>total day SED ↑<br>total day LPA ↑<br>total day MVPA ↑<br><br>during school SED ↑<br>during school LPA ↑<br>during school MVPA ↑<br><br>afterschool SED ↓<br>afterschool LPA ↑<br>afterschool MVPA ↑

**Alhassan et al.; 2016; USA**	RCT	N = 291;<br>4.1 years; ;	141	effects of short bouts of structured PA on preschoolers PA levels	intervent ion	short bouts structured PA	6 months	2x10min/day<br>5 days	TPA<br><br>Accelerometer<br><br>5 days	intervention time<br>total day	$p \leq 0.05$ ↓ LPA, ↑ MVPA<br>total day ↔<br>supporting compensation	overall PA maintained stable<br>higher MVPA is compensated by less LPA	ES (MVPA): d= 0.24<br>ES (LPA): d= 0.15<br>ES (SED): d= 0.21<br>==> small	INT:<br>MVPA ↑* ($p <$ 0.001)<br>LPA ↑* ($p <$ 0.001)<br><br>total day PA ↔
			n.g.											
			n.g.											
			150			no intervention							CON:<br>MVPA ↓<br>LPA ↑<br>SED ↓<br><br>total day PA ↔	
**Alhassan et al.; 2018; USA**	RCT	N = 32 (all f); 8.3 years	14	efficacy of a mother-daughter intervention in girls PA level	intervent ion	CH-M: afterschool dance intervention with mother	12 weeks	60min/day<br>3 times a week	VPA<br>MVPA<br><br>Accelerometer<br><br>7 days	intervention time<br>total afterschool time<br>total day	total day ↔<br>supporting compensation	overall MVPA maintained stable	INT 1:<br>total day SED ↑<br>total day LPA ↓<br>total day MVPA ↔<br><br>afterschool SED ↔<br>afterschool LPA ↓<br>afterschool MVPA ↑	
			12			CH: afterschool dance intervention without mother					total day $p >$ 0.05<br>supporting compensation	overall PA maintained stable<br>PA did not significant change	INT 2:<br>total day SED ↑<br>total day LPA ↓<br>total day MVPA ↑<br><br>afterschool SED ↑<br>afterschool LPA ↑	

											afterschool<br>MVPA ↑
			n.g.								
			6			no intervention					CON:<br>total day SED ↓<br>total day LPA ↑<br>total day MVPA<br>↑<br><br>afterschool SED<br>↓<br>afterschool LPA<br>↑<br>afterschool<br>MVPA ↔
**Aljuhani et al.; 2019; Saudi-Arabien**	Cross Sectional Study	N = 111 (all m); 13.6 years		contribution of PE to daily PA by comparing daily levels on days with and without PE	Physical Education		MVPA<br><br>Accelerometer<br><br>7 days	Day no PE<br>PE day+<br>PE day-	p ≤ 0.05 not supporting compensation	higher PA on PE day than on non-PE day	MVPA<br>PE+ >* non PE-day<br>PE+ > PE-<br>PE- = non PE-day

**Baggett et al.; 2010; USA**Trial of Activity for Adolescent Girls (TAAG)	Cross Sectional Study	N = 6916 (all f); 14.0 years;	evidence of compensation in middle school girlsrelations between inactivity, LPA, MVPA and total PA	daily PA pattern	inactivityLPA MVPAtotal PA Accelerometer 6 days	n.i.	p ≤ 0.05 not supporting compensation	Higher inactivity is associated with lower PA levelsHigher MVPA is associated with higher PA levels	Associations* (p< 0.01) within a day and between two following days↑ inactivity: ↓ LPA ↓ MVPA ↓ total PA↑ MVPA: ↑ LPA ↑ MVPA ↓ inactivity
**Bin Tan et al.; 2019; Singapore**	Cross Sectional Study	N = 5600; 7-18 years	effect of morning commuting on overall walking activity	active commuting to school	step counts<br><br>Pedometer<br><br>6 days	total day morning commuting	p > 0.05 before and after school<br>total day steps Public transport = WALK = CAR supporting compensation	differences in steps before school and after school are compensated at other times	steps before school<br>Public Transport > CAR<br>WALK > CAR<br><br>steps total day<br>Public Transport = WALK = CAR<br><br>steps after school<br>Public transport > CAR<br>WALK > CAR

**Bringolf-Isler et al.; 2009; Switzerland**	Cross Sectional Study	N = 169; 6-14 years; m = 81; f = 88	activity type and quantification of intensity and duration of children's everyday PA	daily PA pattern	MPA<br>VPA<br>MVPA<br>Total PA<br><br>Accelerometer<br><br>7 days	screen rereaction time<br>quit activity<br>public transport<br>recess<br>moderate intense play<br>walking<br>vigorous intense play<br>PE class<br>sport training	p ≤ 0.05 ↑ high intensity sports, ↓ VPA supporting compensation	high intensity sports are compensated by being less vigorous active and more sedentary	Adolescents<br>↑* high intensity sports (p≤ 0.001)<br>↓* playing vigorous (p≤ 0.04)<br>↑* SED (p≤ 0.001)<br>↓* CPM (p≤ 0.007)		
**Brockman et al.; 2010; UK**	Cross Sectional Study	N = 747; 10-11 years; m = 341; f = 406	contribution of active play to children's PA	Active play	MVPA<br>counts per minute<br><br>Accelerometer<br><br>5 days	Weekday<br>after school<br>Weekend	p ≤ 0.05 not supporting compensation	more active play leads to higher PA levels and CPM	Weekday girls<br>MVPA/day ↑* (p≤ 0.01)<br>MVPA/afterschool ↑* (p≤ 0.04)<br>CPM/day ↑* (p≤ 0.01)<br>CPM/afterschool ↑* (p≤ 0.02)<br><br>Weekday boys<br>MVPA/day ↑<br>MVPA/afterschool ↑<br>CPM/day ↑* (p≤ 0.01)<br>CPM/afterschool ↑* (p≤ 0.01)	Weekend girls<br>MVPA/day ↑<br>CPM/day ↑<br><br>Weekend boys<br>MVPA/day ↑<br>CPM/day ↑* (p≤ 0.01)	Whole week girls<br>MVPA/day ↑* (p≤ 0.02)<br>CPM/day ↑* (p≤ 0.01)<br><br>Whole week boys<br>MVPA/day ↑<br>CPM/day ↑* (p≤ 0.01)
**Brusseau et al.; 2011; USA**	Cross Sectional Study	N = 363; 9.7 years; m = 160; f = 203	PA on PE days and non PE days	Physical Education	Step counts<br><br>Pedometer<br><br>7 days	PE day<br>non PE day<br>Weekend day<br>All day	p ≤ 0.05 not supporting compensation	overall PA on PE day is higher than on non PE day	PA<br>PE day >* non PE day (p<0.01)		

**Carlson et al.; 2017; USA**<br><br>Teen Environment and Neighborhood (TEAN) study	Cross Sectional Study	N = 528; 14.12 years; m = 264; f = 264	Association between PA in different locations	PA across locations	MVPA<br><br>Accelerometer<br><br>7 days	home<br>home neighborhood<br>school<br>school neighborhood<br>other	partial compensation<br>$p \leq 0.05$ ↑ PA at school, ↓ PA at other locations supporting compensation	higher at school PA is compensated by less PA at other locations	Associations across location<br>↑Home neighborhood --> at home ↑* (p≤ 0.001)<br>Generalization<br>↑home neighborhood --> school environment ↔<br>↑home neighborhood --> other locations ↑<br>↑at school --> home neighborhood ↓<br>Compensation<br>↑at school --> school environment ↑<br>↑at school --> other locations ↓* (p≤ 0.006)<br>Compensation<br>↑at home --> school environment ↓<br>↑other locations --> school neighborhood ↔<br>↑other locations --> at home ↔
**Cheung et al.; 2019; Hong Kong**	Cross Sectional Study	N = 242; 8.7 years; m = 131; f = 111	associations between school based PA lessons and afterschool hours	PE and afterschool sports classes	MVPA<br><br>Accelerometer<br><br>4 days	afterschool hours<br>school hours	$p \leq 0.05$ not supporting compensation	overall PA is on PE day and Afterschool Sports Program higher than on non active day	N-Act day <* PE = ASP (p≤ 0.00)

**Coombes et al.; 2016; UK**<br><br>Beat the Street - Study	quasi experim ental design	N = 80; 8-10 years	51	impact on active travel to school intervention on increasing active travel	intervent ion	Beat the Street Challenge	9 weeks		MVPA<br><br>Accelerometer<br><br>7 days	total day<br>morning commute<br>afterschool commute<br>evening<br>weekend day	p ≤ 0.05 supporting compensation	higher CPM during active commuting is compensated by less CPM during the evening	CPM/MVPA morning commute WALK >* CAR (p≤ 0.001)<br>CPM/MVPA afternoon commute WALK >* CAR (p≤ 0.03)<br>CPM/MVPA evening WALK <* CAR (p≤ 0.004)	
			n.g.											
			n.g.											
			29			no intervention								
**Cooper et al.; 2003; UK**	Cross Sectiona l Study	N = 114; 10.4 years; m = 59; f = 55		Comparison of PA patterns of children who walked with those who were driven to school	active commuti ng to school				MVPA<br><br>Accelerometer<br><br>7 days	total day<br>8-9 am<br>Weekend	total MVPA Walk = CAR supporting compensation in girls<br><br>p≤ 0.05 not supporting compensation in boys	overall PA is similiar in girls girls compensated for higher PA during 8-9am with lower PA levels at other times<br><br>overall PA is higher in boys who walk compared to boys who were driven	afterschool MVPA WALK >* CAR (p≤ 0.017) only boys during evening MVPA WALK >* CAR (p≤ 0.017) only boys 8-9 am WALK >* CAR (p≤ 0.05)<br><br>total MVPA boys WALK > CAR --> no compensation total MVPA girls WALK = CAR --> compensation	

**Dale et al.; 2000; USA**	Cross Sectional Study	N = 78; 9.3 years; m = 40; f = 38		compensation for a school day on restricted PA by increasing their activity levels after school and during evening	daily PA pattern				Counts per minute<br><br>Accelerometer<br><br>4 days	in school<br>Recess<br>Lunch<br>PE<br>afterschool<br>total day	p ≤ 0.05 not supporting compensation	overall PA is higher on active day than on restricted day	Active day >* restricted day (p≤ 0.01)	
**Eyre et al.; 2015; South Asia**	quasi experimental design	N = 85; INT= 9.5 CON= 11.1 years; m = 40; f = 45	55	effect of integrated school based curriculum and pedometer intervention on PA levels	intervention	pedometer challenge: achieve total daily 18,000 steps	6 weeks		step counts<br><br>Pedometer<br><br>7 days	total day<br>weekday<br>weekend<br>school time<br>after school	p ≤ 0.05 not supporting compensation for INT	INT increased overall PA	INT<br>daily PA ↑* (p≤ 0.001)<br>week PA ↑* (p≤ 0.001)<br>weekend PA ↑<br>school PA ↑* (p≤ 0.01)<br>afterschool PA ↑* (p≤ 0.01)	
			n.g.											
			n.g.											
			30			no intervention					daily PA ↔ (week ↓, weekend ↑) supporting compensation for CON	CON compensated for higher weekend PA by less week PA	CON<br>daily PA ↔<br>week PA ↓<br>weekend PA ↑<br>school PA ↔<br>afterschool PA ↓	

**Ford et al.; 2007; UK**	Cross Sectional Study	N = 239; 8.1 years; m = 125; f = 114	Comparison of daily activity levels in school children who walk with those who travel by car to and from school	active commuting to school	Counts per minute<br><br>Accelerometer<br><br>5 days	weekday<br>in school<br>out of school<br>to and from school commuting time	CPM Walk = CAR supporting compensation	higher to and from school CPM is compensated at other times<br>overall CPM are similar between WALK and CAR	Weekday WALK = CAR<br>In school WALK < CAR<br>out of school WALK < CAR<br>to and from school WALK >* CAR (p≤ 0.000)	
**Fremeaux et al.; 2011; UK**<br><br>Early Bird 46 – Study	longitudinal study	N = 206; 9 years; m = 115; f = 91	effects of different amounts of PA in school on total PA level	Physical Education	total PA<br>MVPA<br><br>Accelerometer<br><br>7 days	in school<br>out of school	TPA S1 = S2 = S3 supporting compensation	overall PA is similiar between the three schools<br>higher PE amounts are compensated	TPA<br>S1 = S2 = S3<br><br>TPA in school<br>S1 >* S2 = S3 (p<0.001)<br><br>TPA out of school<br>S1 < S2 = S3	MVPA<br>S1 = S2 = S3<br><br>MVPA in school<br>S1 >* S2 = S3 (p<0.001)<br><br>MVPA out of school<br>S1 < S2 = S3
**Frömel et al.; 2012; Poland**	Cross Sectional Study	N = 497 (all f); 17.9 years	differences in PA and inactivity structure in girls who do not participate in any organized PA and girls who regulary participate in PE or training lessons	organized sports participation	steps per day<br>LPA<br>MPA<br>VPA<br><br>Accelerometer<br><br>7 days	School<br>Transport<br>Leisure<br>Home<br>total	p ≤ 0.05 not supporting compensation	higher sports club participation is associated with higher overall PA	girls with highest Participation<br>↑* steps/day (p = 0.000)<br>↑* MPA (p = 0.000)<br>↑* VPA (p = 0.000)<br>↑* leisure PA (p = 0.000)	

**Gao et al.; 2015; China**	Cross Sectional Study	N = 68; 10.45 years; m = 28; f = 40		Characterization of children's PA level during a segmented school day	daily PA pattern			Steps per day<br><br>Pedometer<br><br>4 days	total day<br>before school<br>first-fifth session<br>first-third recess<br>lunch break<br>after school	$p \leq 0.05$ not supporting compensation	High active children are more active in every segment than low active children	Boys<br>in recess (high active) HA >* (low active) LA ($p \leq 0.05$)<br>in lunch break HA > LA<br>in PE class HA > LA<br>before school HA >* LA ($p \leq 0.05$)<br>after school HA >* LA ($p \leq 0.05$)	Girls<br>in recess HA >* LA ($p \leq 0.007$)<br>in lunch break HA < LA<br>in PE class HA > LA<br>before school HA > LA<br>after school HA >* LA ($p \leq 0.001$)
**Gidlow et al.; 2008; UK**<br><br>CHAMPS - Study	Cross Sectional Study	N = 503; 10.4 years; m = 250; f = 253		Comparison of in and out PA	daily PA pattern			Counts per minute<br><br>Accelerometer<br><br>7 days	in school<br>out of school<br>school (before and after school weekday)<br>School related PA before, during or immediately after school<br>After school (after 16h)<br>Weekend periods	$p \leq 0.05$ supporting compensation	higher in school CPM are compensated by lower out of school CPM	in school >* out of school ($p \leq 0.001$)	

**Grasten et al.; 2015; Finland**<br><br>Physical Activity as Civil Skill Program	longitudinal study	N = 76; 11.43 years; m = 36; f = 40		Effects of Civil Skill Program on children's LPA patterns	intervention	psychological and physical school environment	2 years		LPA<br><br>Accelerometer<br><br>7 days	Weekend<br>before school<br>After school<br>PE classes<br>Long breaks<br>Short breaks	ES: medium, total LPA ↔ supporting compensation	overall LPA maintained stable LPA is rearranged	boys:<br>total LPA ↔<br>before school LPA ↓<br>after school LPA ↔<br>short break LPA ↔<br>class time LPA ↑<br>long break LPA ↓<br>weekend LPA ↓<br><br>girls:<br>total LPA ↔<br>before school LPA ↓<br>after school LPA ↑<br>short break LPA ↔<br>class time LPA ↑<br>long break LPA ↔<br>weekend LPA ↓
**Grasten et al.; 2015b; Finland**<br><br>Physical Activity as Civil Skill Program	longitudinal study	N = 76; 11.43 years; m = 36; f = 40		Effects of Civil Skill Program on children's MPA patterns	intervention	psychological and physical school environment	2 years		MVPA<br>Accelerometer<br>7 days	Weekendbefore schoolAfter schoolPE classesLong breaksShort breaks	ES: small, total MVPA ↔ supporting compensation	overall MVPA maintained stable MVPA is rearranged	boys:total MVPA ↔before school MVPA ↓after school MVPA ↑short break MVPA ↓class time MVPA ↑long break MVPA ↓weekend MVPA ↑girls:total MVPA ↔before school MVPA ↓after school MVPA ↑short break MVPA ↓class time MVPA ↔long break MVPA ↓weekend MVPA ↓

**Groffik et al.; 2020; Poland/ Czechia**	Cross Sectional Study	N = 921; 16.2 years; m = 324; f = 597		Comparison of two differences education systems of children's PA level	Physical Education			Step counts<br>Pedometer<br>7 days	school time<br>out of school<br>total day	total daily steps CZ = PL, supporting compensation	overall steps are similar in CZ and PL higher PE amounts are compensated	total daily steps CZ = PL
**Goodman et al.; 2011; UK**	Cross Sectional Study	N = 345; 8-13 years; m = 161; f = 184		compensatory effects of different behaviors	daily PA pattern			MVPA<br>Accelerometer<br>4 days	Weekend/week day<br>Own/friend's other home<br>School lesson<br>PE<br>2School Breaks<br>Non home events<br>Clubs<br>Passive travel<br>School/non school active travel<br>Structured sport<br>Out of home play<br>other	↑ inactivity at home, ↑ MVPA during rest of the day small partial supporting compensation<br>p ≤ 0.05 ↑ PE, ↑ MVPA not supporting compensation	higher inactivity at home is compensated by more MVPA during the rest of the day	↑time at home --> ↓ MVPA (p≤ 0.05)<br>↑ time in school --> ↓ MVPA (p≤ 0.05)<br>↑ PE/games, school breaks, active travel, structured sports --> ↑ MVPA (p≤ 0.05)<br>↑at home on weekends --> ↑ MVPA during rest of the day (only partial compensation)
**Haapala et al.; 2017; Finland**<br>Move Program	quasi experimental design	N = 185; 9.3 years	4 school	effects of Move program on children's PA levels	intervention	Move program to make school days more active	2 years	MVPA<br>SED<br>Accelerometer<br>7 days	school day<br>outside school hours<br>total day	p ≤ 0.05 ↑ school MVPA, ↓leisure, ES: small supporting compensation	overall PA maintained stable higher school day PA is compensated by less leisure time PA	School day MVPA ↑* (p = 0.01)<br>School day SED ↓* (P = 0.008)<br>Leisure time MVPA ↓ (p < 0.001)<br>whole day MVPA or ST ↔
			n.g.									

			n.g.											
			2 schools											
**Hardman et al.; 2009; UK**	Cross sectional Study	N = 95; 9.9 years; m = 38; f = 57		Comparison of pedometer steps during school and leisure time	PA during school and Leisure time				step counts<br><br>Pedometer<br><br>6 days	school time leisure time weekday weekend	p ≤ 0.05 not supporting compensation		high and mid active leisure time steps >* school time steps (p < 0.001)<br><br>low active leisure time steps = school time steps	school activity HA >* LA (p < 0.001) HA >* MD (p < 0.001) MD > LA
**Harrington et al.; 2008; UK**<br><br>Girls' Active Program	RCT	N = 1211; 12.8 years		Effects of Girls Active program	intervention	Girls Active Program (framework for school PE)	13 months		LPA MVPA SED<br><br>Accelerometer<br><br>7 days	school hours out of school hours	p ≤ 0.05 not supporting compensation at 7 months<br><br>LPA, MVPA, SED ↔ supporting compensation at 14 months	7 months: overall PA increased<br><br>14 months: overall PA maintained stable (compared to baseline)	7 months: total PA ↑* (p= 0.03) SED during after school ↓* (p= 0.026) total LPA ↑* (p= 0.018) LPA on school day ↑* (p= 0.038)	14 months: no differences at any time of the day

**Herman et al.; 2014; Canada**<br><br>Quebec Adipose and Lifestyle Investigation in Youth (QUALITY) - Study	Cross Sectional Study	N = 532; 9.6 years; m = 286; f = 246		associations between PA intensities	daily PA pattern				LPA<br>VPA<br>MVPA<br>SED<br><br>Accelerometer<br><br>7 days	n.i.	p ≤ 0.05 not supporting compensation	higher PA is associated with higher overall PA and less SED	higher MVPA --> ↓* SED (p < 0.001)<br>higher MVPA --> ↑* TPA (p < 0.001)<br><br>higher VPA --> ↓* SED (p < 0.001)<br>higher VPA --> ↑* TPA (p < 0.001)<br><br>higher LPA --> ↓* SED (p < 0.001)	
**Jago et al.; 2014; UK**<br><br>Action 3:30 - Study	Cross Sectional Study	N = 469; 10.05 years; m = 201; f = 268		differences in PA in those who travelled to school actively compared (A) with those who travelled passively (P)	intervention				Counts per minute MVPA<br><br>Accelerometer<br><br>5 days	total day afterschool hours	p ≤ 0.05 not supporting compensation in girls<br><br>weekday and afterschool MVPA A = P supporting compensation in boys	active travel girls had higher PA levels than passiv travel girls<br><br>active travel boys had similar PA levels than passive travel boys	Travel to school boys<br>Weekday MVPA A = P<br>afterschool MVPA A = P<br><br>Travel from school boys<br>Weekday MVPA A = P<br>afterschool MVPA A = P<br><br>Travel to school girls<br>Weekday MVPA A >* P (p = 0.004)<br>afterschool MVPA A > P<br><br>Travel from school girls<br>Weekday MVPA A > P<br>afterschool MVPA A > P	

**Jago et al.; 2017; UK**<br><br>B-Proact1v - Study	Cross Sectional Study	N = 1223; 9.03 years; m = 556; f = 667	extent to which participation in organized PA in the school or community outside school hours and playing with friends near the home in garden were associated with PA levels	organized sports participation		MVPA<br>SED<br><br>Accelerometer<br><br>5 days	Sport club at school<br>Sport club outside school<br>Plays with friends/family outside near home<br>Plays with friends/family in home or garden	p ≤ 0.05 not supporting compensation	higher frequency of sports participation is associated with higher PA levels	increasing frequency of attending sport/exercise clubs either at school or outside of school and increasing frequency of playing with friends/family outside near the home --> ↓* SED ($p < 0.05$)<br><br>reduction in SED --> ↑* overall activity ($p < 0.05$)<br><br>increasing frequency of attending sport/exercise clubs either at school or outside of school and of play either outside or in home/garden --> ↑* MVPA ($p < 0.05$)
**Kek et al.; 2019; New Zealand**<br><br>Built Environment and Active Transport to School (BEATS) - Study	Cross Sectional Study	N = 314; 14.7 years; m = 103; f = 211	relationship between transport to school habits and PA levels	active commuting to school		MVPA<br><br>Accelerometer<br><br>7 days	total day<br>1h before school<br>1h after school<br>late afterschool hours	p ≤ 0.05 not supporting compensation	active transport (AT) is associated with higher overall PA levels<br><br>Motorized transport (MT)	MVPA on school days<br>AT >* MT ($p<0.05$)<br>AT+MT >* MT ($p<0.05$)<br><br>hour before/after school<br>AT >* AT+MT ($p<0.05$)<br>AT >* MT ($p<0.05$)<br><br>late afterschool hours

												AT = AT+MT = MT
**Kidokoro et al.; 2019; Japan**	quasi experimental design	N = 38; 11.3 years; m = 22; f = 16	18	effects of standing desks on children's PA level	intervention	individual height adjustable standing desks	6 months	LPA<br>MVPA<br>SED<br><br>Accelerometer<br><br>5 days	every hour	p ≤ 0.05, ES: large not supporting compensation	INT increased overall PA	INT group<br>SB ↓* (p< 0.082)<br>LPA ↔<br>MVPA ↑* (p < 0.005)<br><br>overall SED ↓* (p < 0.05)
			n.g.									
			n.g.									

			20			no intervention					
**Kippe et al.; 2018; Norway**	Cross Sectional Study	N = 244; 4-6 years; m = 125; f = 119		children's Pa level at kindergarten related to leisure time	daily PA pattern		MVPA<br><br>Accelerometer<br><br>7 days	preschool time leisure time	p ≤ 0.05 not supporting compensation	higher PA in kindergarten is associated with higher PA levels in leisure time	PA in kindergarten >* PA in leisure time (p < 0.05)<br><br>↑ PA in Kindergarten --> ↑ PA in leisure time
**Kobel et al.; 2017; Germany**<br><br>Join the Healthy Boat – Study	Cross Sectional Study	N = 294; 7.1 years; m = 141; f = 153		effects of PE on children's PA levels	Physical Education		MVPA<br><br>Accelerometer<br><br>6 days	PE day<br>non PE day	p ≤ 0.05 not supporting compensation	overall PA is higher on PE days than on non-PE days	MVPA<br>PE days >* no PE day (p ≤ 0.01)<br><br>PE day - PA during PE > non PE day
**Koorts et al.; 2019; Australia**<br><br>NEighbourhood Activity in Youth (NEArbY) – Study	Cross Sectional Study	N = 358; 15.3 years; m = 146; f = 212		Contribution of sports participation to overall PA	organized sports participation		MVPA<br><br>Accelerometer<br><br>8 days	n.i.	p ≤ 0.05 not supporting compensation	higher frequency of sports participation is associated with higher PA levels	↑ number and frequency of sports participation --> ↑* MVPA (p=0.004)

**Long et al.; 2013; USA**<br><br>NHANES - Study	Cross Sectional Study	N = 2548; 9.5 years; m = 1266; f = 1282		effects of higher school day PA on overall PA	daily PA pattern				VPA<br>MVPA<br><br>Accelerometer<br><br>7 days	before school<br>school time<br>after school<br>evening	p ≤ 0.05 not supporting compensation	higher school day PA is associated with higher overall PA	1 additional min of school day MVPA --> 1.14min of total day MVPA* (p < 0.001)<br><br>1 additional min of school day VPA --> 1.15min of total day VPA* (p < 0.001)<br><br>higher school day MVPA --> ↑ total daily MVPA
**Loucaides et al.; 2009; Cyprus**	RCT	N = 247; 11.1 years	89	effects of interventions on children's PA	intervention	play space for team games, playground markings and long ropes	2 months		Step counts<br><br>Pedometer<br><br>4 days	20min break<br>after school	p ≤ 0.05, ES: small not supporting compensation	INT increased mean overall steps	INT 1:<br>4 weeks after intervention mean steps ↑* (p < 0.001; p < 0.01)<br><br>afterschool activity ↑
			89			play spaces for team games	2 months					INT increased mean overall steps	INT 2:<br>4 weeks after intervention mean steps ↑* (p < 0.001; p < 0.01)<br><br>afterschool activity ↑
			n.g.										
			69			no intervention							

**Magnusson et al.; 2011; Iceland**	Cluster RCT	N = 196; 7.3 years; m = 87; f = 109	100	changes in volume and intensity of PA	intervention	school based PA intervention (engage children to be more physically active during school)	2 years		counts per minute<br>MVPA<br><br>Accelerometer<br><br>7 days	in school<br>out of school	$p \leq 0.05$ not supporting compensation during first year of intervention<br><br>INT = CON supporting compensation at the end of the intervention	first year:<br>higher PA levels in INT compared to CON<br><br>End of intervention: INT and CON had similar PA levels	after 1 year during school hours PA<br>INT >* CON ($p < 0.0001$)<br><br>end of intervention during school hours PA<br>INT = CON<br><br>after 1 year after school hours PA<br>INT > CON<br><br>end of intervention after school hours PA<br>INT = CON
			n.g.										
			n.g.										
			96			no intervention							
**Mallam et al.; 2003; UK**	Cross Sectional Study	N = 159; 9.0 years; m = 85; f = 74		impact of timetabled PE on overall PA	Physical Education				units/week<br><br>Accelerometer<br><br>7 days	total day<br>in school<br>after school	$p \leq 0.05$ ↓ out of school, ↑ in school<br>TPA S1 = S2 = S3 supporting compensation	overall PA is similiar between the three schools higher PE amonunts are compensated	TPA<br>S1 = S2 = S3<br><br>TPA in school<br>S1 >* S2 = S3 ($p > 0.001$)<br><br>TPA out of school<br>S1 <* S2 = S3 ($p > 0.001$)

**Mayorga-Vega et al.; 2019; Spain**	Cross Sectional Study	N = 158; 14.6 years; m = 83; f = 75	Comparison PA level on PE days and non PE days	Physical Education	LPA<br>MVPA<br>step counts<br><br>Accelerometer<br><br>7 days	PE day<br>non PE day<br>Weekend<br>PE lesson	p ≤ 0.05, ES: medium not supporting compensation	overall PA is higher on PE day compared to non-PE day	TPA<br>PE days >* non PE days (p< 0.001)<br><br>MVPA<br>PE days >* non PE days (p< 0.05)<br><br>LPA<br>PE days >* non PE days (p< 0.001)<br><br>SED<br>PE days <* non PE days (p< 0.01)
**Meyer et al.; 2013; Switzerland**<br><br>Kinder- und Jugendsport-Study (KISS)	Cross Sectional Study	N = 676; 9.3 years; m = 329; f = 347	Effect of PE on PA level	Physical Education	MVPA<br>total PA<br><br>Accelerometer<br><br>7 days	PE day<br>non PE day<br>PE day without PA in PE lesson	p ≤ 0.05 not supporting compensation	overall PA is higher on PE day compared to non-PE day	TPA<br>PE days >* non PE days (p< 0.001)<br><br>MVPA<br>PE days >* non PE days (p< 0.001)

**Møller et al.; 2014; Denmark**<br><br>The CHAMPS - Study DK	quasi experimental design	N = 2130; 9,9 years		Effects of sport school on children PA level in different domains	intervention	increased mandadory PE lessons from 2 to 6 lessons per week	1 year	at least 4,5h PE/week	MVPA<br>SED<br><br>Accelerometer<br><br>7 days	school time<br>leisure time	$p \leq 0.05$ ↑ school time PA in sport schools, ↓ leisure time PA in sports schools<br>total PA sport schools = normal schools<br>supporting compensation	higher school time PA in sports schools is compensated by less leisure time PA in sports school<br>overall PA is similar between sports and normal schools	school time PA<br>boys in sport schools >* boys in normal schools ($p < 0.001$)<br>girls in sport schools > girls in normal schools<br><br>leisure time MVPA<br>boys in sport schools <* boys in normal schools ($p < 0.001$)<br>girls in sport schools <* girls in normal schools ($p < 0.004$)<br><br>leisure time SED<br>boys in sport schools > boys in normal schools<br>girls in sport schools > girls in normal schools<br><br>total PA, PE, recess<br>children in sport schools = children in normal schools
**Mosses et al.; 2019; Estonia**	Cross Sectional Study	N = 492; 9.3 years; m = 226; f = 266		contributing role of organized sport participation to daily MVPA	organized sports participation				MVPA<br><br>Accelerometer<br><br>7 days	during training<br>outside of training	$p \leq 0.05$ not supporting compensation	overall PA is higher on training day than non training day<br>more training PA is associated with increased overall PA	1 additional min of training MVPA --> 1.3 min increase in daily MVPA<br><br>MVPA<br>training day >* non training day -($p< 0.05$)

**Morgan et al.; 2007; USA**	Cross Sectional Study	N = 485; 6-11 years; m = 205; f = 280	contribution of PE to daily PA by comparing daily levels on days with and without PE	Physical Education	step counts<br><br>Pedometer<br><br>2 weeks	PE day<br>non-PE day	$p \leq 0.05$ not supporting compensation	overall PA is higher on PE day compared to non-PE day	steps/day high active group<br>PE day >* non PE day ($p < 0.001$)<br><br>steps/day moderate active group<br>PE day >* non PE day ($p < 0.001$)<br><br>steps/day low active group<br>PE day >* non PE day ($p < 0.001$)	

**Nielsen et al.**<br><br>COSCIS - Study	Cross Sectional Study longitudinal study	2001: N = 594; 6.3 years; m = 310, f = 284<br><br>2004: N = 518; 9.5 years; m = 266, f = 252		association between the number of available school ground play facilities and daily PA	school playground facilities / active play				MPA<br>VPA<br>MVPA<br><br>Accelerometer<br><br>5 days	during school<br>outside school hours<br>total day	p ≤ 0.05 not supporting compensation	more play facilities increase overall PA	Preschool children<br>each 10 additional play facilities --> ↑* PA during school time (p < 0.001)<br>each 10 additional play facilities --> ↑* PA overall (p < 0.001)<br>each 10 additional play facilities --> ↑* MVPA (p < 0.001)<br>each 10 additional play facilities --> ↑* VPA (p = 0.002)	school children<br>each 10 additional play facilities --> ↑* PA during school time (p < 0.001)<br>each 10 additional play facilities --> ↑* PA overall (p < 0.001)<br>each 10 additional play facilities --> ↑* MVPA (p < 0.001)<br>each 10 additional play facilities --> ↑* VPA (p = 0.003)
**O'Dwyer et al.; 2013; UK**	Cluster RCT	N = 156; 4.5 years; m = 86; f = 70	70	effect of school based active play intervention on children's PA level	intervention	preschool based active play	6 weeks	60min/day<br>1 time a week	MVPA<br>SED<br><br>Accelerometer<br><br>7 days		p ≤ 0.05 ↑ active play session<br>total day INT = CON supporting compensation	increased PA during active play session is compensated by less PA at other times overall PA is similar between INT and CON	active play session<br>INT >* CON (p< 0.001)<br><br>Total day<br>INT = CON	

			n.g.								
			n.g.								
			86			no intervention					
**O'Neill et al.; 2016; USA**	Cross sectiona l Study	N = 341; 4.6 years; m = 174; f = 167		Relationship between PA in preschool or child care center setting and their activity levels outside this setting	Prescho ol PA		total PA<br>MVPA<br><br>Accelerometer<br><br>5 days	in preschool<br>out of preschool<br>total day	p ≤ 0.05 not supporting compensation	higher in school PA is associated with higher out of school PA	MVPA and total PA<br>out of school >* in school (p < 0.01)<br><br>Association<br>↑in school -->* ↑out of school (p < 0.02 MVPA; p < 0.01 TPA)

**Owen et al.; 2003; UK**<br><br>Child Heart and Health - Study (CHASE)	Cross sectiona l Study	N = 2035; 9.9 years; m = 974; f = 1061	impact from changing from car to more active forms of travelling on PA levels	ACS	step counts<br>counts per minute<br>LPA<br>MPA<br>VPA<br>MVPA<br>SED<br><br>Accelerometer<br><br>7 days	travel period<br>out of travel period	$p \leq 0.05$ not supporting compensation	public transport and walk/cycle had overall higher PA levels than car children	travel time:<br>weekday activity counts<br>Car* < walk/cycle ($p< 0.01$)<br>public transport = walk/cycle<br><br>steps/day<br>public transport >* Car ($p< 0.0001$)<br>walk/cycle = public transport<br><br>MVPA<br>Car <* walk/cycle ($p< 0.001$)<br>public transport > walk<br><br>periods outside travel time:<br>steps/day<br>car < walk/cycling<br>public transport > walk/cycle<br><br>MVPA<br>car < walk/cycle<br>public transport > walk/cycle

**Palmer et al.; 2019; USA**<br><br>CHAMP - Study	Cluster RCT	N = 61; 4.41 years; m = 40; f = 21	44	changes in motor skills and PA through the intervention	intervention	motor skill intervention	5 weeks	600min<br>40 min/day<br>3 times a week	LPA<br>MPA<br>VPA<br>MVPA<br>SED<br>total PA<br><br>Accelerometer<br><br>7 days	intervention day<br>outdoor free play	p ≤ 0.05 ↑ MVPA, ↓ LPA, ES: medium<br>TPA ↔<br>supporting compensation	overall PA maintained stable higher MVPA is compensated by less LPA	during CHAMP at the end of intervention<br>↓* LPA ($p < 0.005$)<br>↑* VPA ($p < 0.004$)<br>↑* MVPA ($p < 0.003$)<br>↔ TPA<br><br>during outdoor free play<br>no changes
			n.g.										
			n.g.										
			17			no intervention							

**Pate et al.; 2016; USA**<br><br>Study of Health and Activity in Preschool Environments (SHAPES)	RCT	N = 379; 4.5 years; m = 191; f = 188	188	effects of an adaptable ecologic preschool intervention on PA level	intervention	Engagement of children's PA in (1) structured, teacher-led physical activity opportunities in the classroom (2) structured and unstructured physical activity opportunities at recess (3) physical activity integrated into pre-academic lessons	1 year	LPA<br>MVPA<br>total PA<br><br>Accelerometer<br><br>5 days	$p \leq 0.05$ ↑ MVPA<br>TPA ↔<br>supporting compensation	overall PA maintained stable higher MVPA is compensated by less LPA	INT:<br>TPA ↔<br>MVPA ↑ (* in girls: $p < 0.04$)<br>LPA ↓
			n.g.								
			n.g.								
			191			no intervention					

**Pelklova et al.; 2010; Czechia**	longitudinal study	N = 13; 15.2 years; m = 2; f = 11		pedometer-determined PA over one school year for two groups: children who engage regular in afterschool PA and those who were not engaged in regular afterschool PA	organized after school sports participation	i				step counts Pedometer 1 year	weekday with PEweekday without PEweekend	$p \leq 0.05$ not supporting compensation	higher overall PA in children who partake in afterschool PA	steps/dayweekdays with PE ASPA >* non ASPA ($p < 0.0001$)steps/day weekdays without PE ASPA >* non ASPA ($p < 0.0001$)steps/day weekendASPA >* non ASPA ($p < 0.0001$)		

**Pesola et al.; 2018; Finland**	Cross sectiona l study	N = 15;<br>10.3 years;<br>m = 7; f = 8		effect of visiting SuperPArk on children's PA levels	SuperPa rk visit (areas for different PA activitie s)				LPA<br>MVPA<br>SED<br><br>Accelerometer<br><br>7 days	whole day<br>SuperPark visit period<br>SuperPark day<br>Reference day	$p \leq 0.05$ not supporting compensation	higher overall PA in childre who visit SuperPark	whole week superpark period<br>sitting: INT <* CON ($p < 0.001$)<br>MVPA: INT >* CON ($p = 0.002$)<br>long sitting: INT <* CON ($p = 0.002$)<br><br>whole week whole day<br>sitting: INT <* CON ($p < 0.008$)<br>MVPA: INT >* CON ($p = 0.0017$)	weekday superpark period<br>sitting: INT <* CON ($p < 0.028$)<br>LPA: INT <* CON ($p = 0.046$)<br>MVPA: INT >* CON ($p = 0.028$)<br>long sitting: INT <* CON ($p = 0.028$)<br><br>weekday whole day<br>LPA: INT <* CON ($p = 0.028$)<br>MVPA: INT >* CON ($p = 0.028$)	weekend superpark period<br>sitting: INT <* CON ($p < 0.018$)<br>MVPA: INT >* CON ($p = 0.028$)

**Ribeyre et al.; 2000; France**	Cross Sectiona l Study	N = 50; 17.0 years; m = 27; f = 23	day to day variations in daily EE of athletic and non athletic adolescents	Daily EE Pattern	EE<br><br>HR monitor<br><br>7 days	total day<br>sleep<br>school work<br>meals<br>Exercise<br>miscellaneous activity	p ≤ 0.05 ↑ EE during exercise, ↓ EE during school and miscellaneous work supporting compensation	athletes compensated for higher EE during exercise with lower EE during school work and miscellaneous work	EE during school work<br>athletes <* non athletes (p < 0.01)<br><br>EE during exercise<br>athletes >* non athletes (p < 0.001)<br><br>EE during miscellaneous work<br>athletes <* non athletes (p < 0.001)
**Ridgers et al.; 2014; Australia**<br><br>Patterns of Habitual Activity across Seasons (PHASE) - Study	Cross Sectiona l Study	N = 248; 10.0 years; m = 121; f = 127	Associations between PA levels on one day with PA levels on the following day	daily PA pattern	LPA<br>MVPA<br>SED<br><br>Accelerometer<br><br>7 days	given day<br>following day	p ≤ 0.05 ↑ PA on day 1, ↓ PA on day 2 supporting compensation	higher PA on one day is compensated by less PA on the other day	MVPA(D1) ---> MVPA(D2) ↓* (p < 0.001)<br>LPA (D1) --> LPA(D2) ↓* (p < 0.001)<br>SED (D1) --> SED(D2)↓* (p < 0.014)<br>LPA (D1) --> MVPA(D2)↓* (p < 0.001)<br>MVPA (D1) --> LPA(D2)↓* (p < 0.001)

**Ridgers et al.; 2015; Australia**<br><br>Patterns of Habitual Activity across Seasons (PHASE) - Study	Cross Sectional Study	N = 235; 10.1 years; m = 125; f = 110		correlations between primary school children's sitting, standing and stepping time within and between days	PA within and between days				sitting time<br>standing time<br>stepping time<br><br>Accelerometer<br><br>8 days	before school<br>at school<br>after school	p ≤ 0.05 ↑ PA on day 1, ↓ PA on day 2 supporting compensation	higher PA on one day (D1) is compensated by less PA on the other day (D2)	Weekday<br>Step(D1) --> Step(D2) ↓* (p < 0.001)<br>Step(D1) --> Stand(D2) ↓* (p < 0.001)<br>Step(D1) --> Sit(D2) ↑<br>Sit(D1) --> Sit(D2) ↓* (p < 0.001)<br>Sit(D1) --> Stand(D2) ↑* (p = 0.004)<br>Sit(D1) --> Step(D2) ↑* (p < 0.001)<br>Stand(D1) --> Stand (D2) ↓* (p < 0.001)<br>Stand(D1) --> Step (D2) ↑<br>Stand(D1) --> Sit(D2) ↑* (p < 0.001)	Weekend day<br>Step(D1) --> Step(D2) ↓* (p < 0.001)<br>Step(D1) --> Stand(D2) ↓* (p < 0.001)<br>Step(D1) --> Sit(D2) ↑* (p > 0.001)<br>Sit(D1) --> Sit(D2) ↓* (p < 0.001)<br>Sit(D1) --> Stand(D2) ↑* (p < 0.001)<br>Sit(D1) --> Step(D2) ↑* (p < 0.001)<br>Stand(D1) --> Stand (D2) ↓* (p < 0.001)<br>Stand(D1) --> Step (D2) ↓* (p < 0.001)<br>Stand(D1) --> Sit(D2) ↑* (p < 0.001)

**Ridgers et al.; 2017; Australia**<br><br>Fitness, Activity and Skills Testing (FAST) - Study	Cross Sectional Study	ACC: N = 127; 10.45 years; m = 64, f = 63<br><br>SW: N = 98; 10.45 years; m = 48, f = 50	Association between Pa levels and EE on one day with these behaviors on the following day	daily PA pattern	LPA<br>MVPA<br>EE<br><br>Accelerometer<br>SenseWear Armband<br><br>8 days	given day<br>following day	$p \leq 0.05$ ↑ PA on day 1, ↓ PA on day 2 supporting compensation	higher PA on one day (D1) is compensated by less PA on the other day (D2)	MVPA(D1) ---> MVPA(D2) ↓* ($p < 0.001$)<br>LPA (D1) --> LPA(D2) ↓* ($p < 0.001$)<br>LPA (D1) --> MVPA(D2)↓* ($p < 0.001$)<br>MVPA (D1) --> LPA(D2)↓* ($p < 0.001$)<br>EE(D1) --> EE(D2) ↓* ($p < 0.001$)

**Ridgers et al.; 2017b; Australia**<br><br>Reactivity to Activity (REACT) – Study	randomized but not controlled Study	ACC: N = 156; 11.3 years; m = 72, f = 84<br><br>SW: N = 145; 11.3 years; m = 71, f = 74		Exploring the ActivityStat hypothesis by investigating children's short term response to imposed or restricted PA	intervention	1. additional MVPA (sport session delivered during morning class - 120min session)<br>2. additional LPA (standing lesson - 120min)<br>3. restricted LPA and MVPA	1 week		LPA<br>MVPA<br>SED<br><br>Accelerometer<br>SenseWear Armband<br><br>5 days	experimental day<br>day after experimental day	$p \leq 0.05$ not supporting compensation	higher PA on one day is associated with no changes in PA on the next day (compared to usual PA on that day)	Increased MVPA (Sport Club Session)<br>↑*SED on day after Exp. ($p< 0.05$)<br>↓*MVPA on day after Exp.($p< 0.05$)<br>↓* CPM on day after Exp.($p< 0.05$)<br><br>But: no differences with usual PA on that day	Increased LPA (Standing lesson Session)<br>no differences with usual PA on that day	Restricted MVPA and LPA (Indoor play)<br>↓*SED on day after Exp. ($p< 0.05$)<br>↑*MVPA on day after Exp. ($p< 0.05$)<br>↑*CPM on day after Exp. ($p< 0.05$)<br>↑*LPA on day after Exp. ($p< 0.05$)<br><br>But: no differences with usual PA on that day<br>↑*EE on day after Exp.
**Rooney et al.; 2018; UK**	Cross Sectional Study	N = 61; 9.3 years; m = 24; f = 37		influence of PE and recess on overall PA	Physical Education				MVPA<br><br>Accelerometer<br><br>4 days	PE day<br>non-PE day<br>PE<br>Recess<br>Lunch	PE day = non PE day supporting compensation	overall PA is similar on PE day and non PE day	PA on PE day = PA on non PE day		

**Schneller et al.; 2017; Denmark**<br><br>TEACHOUT – Study	quasi experimental design	N = 361; 10.9 years; m = 139; f = 222	201	Effect of Education outside the Classroom (EOtC) on PA levels	intervention	EOtC	1 year	300min weekly	LPA<br>MPA<br>VPA<br>MVPA<br><br>Accelerometer<br><br>7 days	n.i.	$p \leq 0.05$ not supporting compensation in boys<br><br>INT = CON supporting compensation in girls	higher overall PA in boys with EOtC<br><br>PA levels similar in girls with EOtC and without EOtC	boys:<br>MVPA, VPA, MPA<br>EOtC >* CON ($p < 0.05$)<br><br>LPA<br>EOtC = CON<br><br>Girls<br>MVPA, VPA, MPA, LPA<br>EOtC = CON
			n.g.										
			n.g.										
			162			no intervention							

**Schneller et al.; 2017b; Denmark**<br><br>TEACHOUT – Study	quasi experimental design	N = 663; 10.8 years		Effect of Education outside the Classroom (EOtC) on PA levels in different domains	intervention	EOtC	1 year	300min weekly	LPA<br>MVPA<br><br>Accelerometer<br><br>7 days	schoolday with EOtC schoolday without EOtC	p ≤ 0.05 not supporting compensation	higher overall PA levels on days with EOtC compared to a day without EOtC	boys:<br>LPA<br>school day with EOtC >* school day without EOtC and PE (p< 0.001)<br>school day with EOtC >* school day with PE (p < 0.001)<br>school day with EOtC >* weekend day (p < 0.001)<br><br>girls:<br>LPA<br>school day with EOtC >* school day without EOtC and PE (p< 0.001)<br>school day with EOtC >* school day with PE (p < 0.001)<br>school day with EOtC >* weekend day (p < 0.001)

**Sigmund et al.; 2014; Czechia**	Cross Sectional Study	N = 338; 9.91 years; m = 168; f = 170	Changes in PA levels between the day with participation in PE and without PE	Physical Education	LPA<br>MVPA<br>SED<br><br>Accelerometer<br><br>7 days	Before school<br>in school<br>after school<br>all day	p ≤ 0.05, ES: medium not supporting compensation	higher overall PA levels on day with PE compared to non PE day	school MVPA<br>PEL >* without PEL (p < 0.005)<br><br>daily MVPA<br>PEL >* without PEL (p girls < 0.05), (p boys < 0.005)<br><br>school LPA<br>PEL >* without PEL (p girls < 0.005), (p boys < 0.01)<br><br>daily SED<br>PEL <* without PEL (p girls < 0.05), (p boys < 0.01)
**Smith et al.; 2012; UK**<br><br>SPEEDY - Study	longitudinal study	N = 812; 9-10 years; m = 333; f = 479	Association between change in mode of travel to school and change in PA	ACS	MVPA<br><br>Accelerometer<br><br>7 days	total day<br>weekday	p ≤ 0.05 not supporting compensation	higher overall PA in children who changed from passive to active travel	boys:<br>change in total daily MVPA<br>active to passive ↓<br>passive to active ↑* (p= 0.03)<br><br>girls:<br>change in total daily MVPA<br>active to pasive ↓<br>passive to active ↑* (p< 0.01)

**Stewart et al.; 2017; New Zealand**<br><br>Built Environment and Adolescent New Zealanders (BEANZ) – Study	Cross sectional study	N = 186; 14.07 years; m = 110; f = 76	Relationship between school travel mode and physical activity within various space-time domains	ACS	counts per minute MVPA Accelerometer 7 days	total day home school transport leisure	$p \leq 0.05$ not supporting compensation	higher overall PA in children with active transport (AT) higher transport PA is associated with higher leisure time PA<br><br>no active transport (noT)	Day MVPA AT >* noT ($p < 0.001$)<br>Day SED AT < noT<br>Home MVPA AT = noT<br>Home SED AT = noT<br>School MVPA AT = noT<br>School SED AT = noT<br>Transport MVPA AT >* noT ($p < 0.001$)<br>Transport SED AT <* noT ($p < 0.001$)<br>Leisure MVPA AT >* noT ($p < 0.039$)<br>Leisure SED AT <* noT ($p < 0.016$)

**Stylaniou et al.; 2016; USA**<br><br>Comprehensive school physical activity program – Study (CSPAP)	alternating Treatment	N = 88; 8-10 years	39	compensation for a before school program on PA at other times	intervention	Before school running/walking club	5 weeks	20 min/day<br>2 times/week	step counts<br>MVPA<br><br>Pedometer<br><br>1 year	during program<br>total day	$p > 0.05$, supporting compensation in school A	School A did not change overall PA levels	before school program participation --> ↑
			49					15 min/day<br>2 times/week			$p \leq 0.05$; ES: small not supporting compensation in school B	School B had higher overal PA levels	before school program participation --> ↑* ($p<0.001$)
			n.g.										
			n.g.										
**Sutherland et al.; 2017; Australia**<br><br>Supporting Children's Outcomes using Rewards, Exercise and Skills - Study (SCORES)	RCT	N = 989; 8-11 years	571	Effectivness of an adapted version of an evidence based school PA program on children's PA	intervention	multicomponent school based intervention	6 months		MPA<br>VPA<br>MVPA<br><br>Accelerometer<br><br>7 days	total day<br>school day<br>recess and lunch<br>PE lesson	$p \leq 0.05$ ↑ school day MVPA, ↓ out of school MVPA<br>daily MVPA INT = CON supporting compensation	higher school day PA is compensated by less out of school PA<br>overall PA is similar between INT and CON	overall daily MVPA<br>INT = CON<br><br>overall VPA<br>INT >* CON ($p= 0.04$)<br><br>school day MVPA<br>INT >* CON ($p= 0.05$)<br><br>school day VPA<br>INT >* CON ($p\leq 0.001$)
			n.g.										

			n.g.										
			568			no intervention							
**Toftager et al.; 2014; Denmark**<br><br>SPACE - Study	Cluster RCT	N = 797; 12.5 years; m = 402; f = 395	376	Effectivness of the intervention in reducing decline in PA	intervent ion	physiological changes and organizational environment	2 years		MVPA<br>total PA<br><br>Accelerometer<br><br>7 days	total day<br>weekday<br>weekend<br>school time<br>recess	overall PA ↔ supporting compensation	overall PA mainated stable	INT:<br>overall PA ↔<br>MVPA ↓<br>school PA ↓<br>Recess PA ↓<br>SED ↑
			n.g.										
			n.g.										
			421			no intervention							CON:<br>overall PA ↓<br>MVPA ↓<br>school PA ↓<br>Recess PA ↓<br>SED ↑

**Trapp et al.; 2013; Australia**<br><br>TRavel, Environment, and Kids – Study (TREK)	Cross sectional Study	N = 884; 11.0 years; m = 384; f = 500	Extent of travel mode to school was associated with PA	ACS	step counts<br><br>Pedometer<br><br>7 days	weekend<br>weekday<br>leisure time	p ≤ 0.05 not supporting compensation	higher overall PA in children who walk compared to children who travelled with car	steps on weekday<br>WALK >* CAR (p < 0.001)<br><br>Participation in leisure activities<br>WALK >* CAR (p < 0.05 for girls; p < 0.001 for boys)	after adjusting for demographic factors<br>Steps<br>WALK >* CAR (p < 0.000)<br><br>Participarion in leisure activities<br>WALK >* CAR (p < 0.043) for girls<br>WALK = CAR
**Tudor Locke et al.; 2006; USA**	Cross sectional study	N = 81; 11.8 years; m = 28; f = 53	Differences in step counts on PE and non PE day in different domains	Physical Education	step counts<br><br>Pedometer<br><br>4 days	Total day<br>Before school<br>After School<br>Lunchtime<br>Recess<br>PE<br>After school<br>Residual	PE day = non PE day supporting compensation in girls<br><br>p ≤ 0.05 not supporting compensation in boys	overall PA levels on day with PE and without PE similar in girls<br><br>higher overall PA levels on PE days compared to non-PE days in boys	boys:<br>steps<br>PE day >* non PE day (p < 0.05)	girls<br>steps<br>PE day = non PE day

**Vale et al.; 2011; Portugal**<br><br>Preschool Physical Activity, Body Composition and Lifestyle - Study (PRESTYLE)	Cross sectional Study	N = 193; 4.8 years; m = 97; f = 96	Differences in PA during daily school hours when they attend PE classes compared with school days without PE classes	Physical Education	total PA<br>MVPA<br><br>Accelerometer<br><br>5 days	PE day<br>non PE day	p ≤ 0.05 not supporting compensation	overall PA levels higher on days with PE compared to non-PE day	TPA<br>PE day >* non PE day (p < 0.05)<br><br>MVPA<br>PE day >* non PE-day (p < 0.05	
**Van Cauwenbergh et al.; 2013; Belgium**	Cross Sectional Study	N = 200; 5.3 years; m = 87; f = 113	Comparison of PA levels on days with Teacher led structured PA session with days without these sessions	teacher led structured PA session	LPA<br>MVPA<br>LMVPA<br>SED<br><br>Accelerometer<br><br>4 days	Preschool time<br>after preschool	p ≤ 0.05 not supporting compensation	higher PA during school day on PE day<br>similar afterschool PA levels on day with and without PE<br><br>higher overall PA levels on day with PE	Preschool time<br>SED<br>PE day >* without (PE p < 0.001)<br><br>LPA<br>PE day >* without PE (p < 0.001)<br><br>MVPA<br>PE day >* without PE (p < 0.001)	After Preschool time<br>SED<br>PE day = without<br><br>LPA<br>PE day = without<br><br>MVPA<br>PE day = without

**Wilson et al.; 2017; Australia**	Cross over design	N = 51 (all m); 12.4 years	impact of 10min active lesson breaks on PA on other day segments and across the whole day	intervention	active school lesson breaks	1 week	40min/day 3 times a week	MPA VPA MVPA Accelerometer 7 days	total day school hours ALB afterschool	$p \leq 0.05$ ↑ in school MVPA, ↓ afterschool MVPA daily MVPA INT = CON supporting compensation	higher inschool PA is compensated in less out of school PA overall PA levels similar between INT and CON	daily MVPA INT = CON<br>daily VPA INT >* CON (p=0.006)<br>daily MPA INT < CON<br>inschool MVPA INT >* CON (p<0.0001)<br>afterschool MVPA INT <* CON (p<0.01)

(n.g. = not given in the study)

Abbreviation List

CON	Control group
CPM	Counts per minute
EE	Energy Expenditure
ES	Effect Size
INT	Intervention group
LPA	Light physical activity
MVPA	Moderate-to-vigorous physical activity
PA	Physical activity
PE	Physical Education
RCT	Randomized controlled trial
SED	Sedentary behavior

ST	Screen time
TPA	Total physical activity
VPA	Vigorous physical activity
