# Supplementary material for: Compensation or Displacement of Physical Activity in Children and Adolescents: A Systematic Review of Empirical Studies
Source: Children (Basel). 2022 Mar 3;9(3):351. doi: 10.3390/children9030351 (PMC8947494; doi:10.3390/children9030351)
Supplement: Supplementary file 1 [file children-09-00351-s001.zip › Table S2 Quality Assessment of included studies.pdf]

Table S2: Quality Assessment of included studies

Study	*Question / objective sufficiently described?*	*Study design evident and appropriate?*	*Method of subject/comparison group selection or source of information/input variables described and appropriate?*	*Subject (and comparison group. if applicable) characteristics sufficiently described?*	*If interventional and random allocation was possible. was it described?*	*If interventional and blinding of investigators was possible. was it reported?*	*If interventional and blinding of subjects was possible. was it reported?*	*Outcome and (if applicable) exposure measure(s) well defined and robust to measurement / misclassification bias? Means of assessment reported?*	*Sample size appropriate?*	*analytic methods described/justified and appropriate?*	*Some estimate of variance is reported for the main results?*	*Controlled for confounding?*	*Results reported in sufficient detail?*	*Conclusion supported by results*	*Summary score (total sum / total possible sum)*
Abuerto 2011	1	2	2	2	2	1	1	2	2	2	2	2	2	2	0.89
Adamo 2017	2	2	2	2	1	2	0	2	2	2	2	2	2	1	0.86
Adkins 2013	1	2	1	2	1	0	0	2	1	2	1	1	1	1	0.57
Alderman 2012	2	2	2	2	n/a	n/a	n/a	2	2	2	1	2	2	2	0.95
Alhassan 2007	2	2	2	1	1	0	0	2	1	2	1	2	1	2	0.68
Alhassan 2012	1	2	2	2	1	0	0	2	1	2	2	2	2	2	0.75
Alhassan 2013	2	2	1	1	1	0	0	2	2	2	1	2	2	2	0.71
Alhassan 2016	2	2	2	2	2	0	0	2	2	2	1	1	2	2	0.79
Alhassan 2018	1	2	2	1	1	0	0	2	2	2	2	1	2	2	0.71
Aljuhani 2019	2	2	2	2	n/a	n/a	n/a	2	2	2	2	2	2	2	1.00

Baggett 2010	2	2	2	2	n/a	n/a	n/a	2	2	2	2	2	2	2	1.00
Bin Tan 2018	2	2	1	1	n/a	n/a	n/a	2	2	2	2	2	2	2	0.91
Bringolf Isler 2009	2	2	2	2	n/a	n/a	n/a	2	2	2	2	2	2	2	1.00
Brockman 2010	1	2	2	1	n/a	n/a	n/a	2	2	2	2	2	1	1	0.82
Brusseau 2011	2	2	2	1	n/a	n/a	n/a	2	2	2	1	1	2	2	0.86
Carlson 2017	2	2	2	2	n/a	n/a	n/a	2	2	2	1	2	2	2	0.95
Cheung 2019	2	2	2	2	n/a	n/a	n/a	2	2	2	1	1	2	2	0.91
Coombes 2016	2	2	2	1	n/a	n/a	n/a	2	2	2	2	2	2	2	0.95
Cooper 2003	1	2	2	2	n/a	n/a	n/a	1	2	1	1	1	2	2	0.77
Dale 2000	2	2	2	1	n/a	n/a	n/a	2	2	2	1	1	1	1	0.77
Eyre 2015	2	2	1	1	n/a	n/a	n/a	2	2	2	2	2	1	2	0.86
Ford 2007	1	2	2	2	n/a	n/a	n/a	2	2	2	2	2	2	2	0.95
Fremeaux 2011	1	2	2	2	n/a	n/a	n/a	2	1	2	1	2	2	1	0.82
Frömel 2012	2	2	2	2	n/a	n/a	n/a	2	2	2	2	1	2	2	0.95
Gao 2015	2	2	2	2	n/a	n/a	n/a	1	1	2	1	1	2	2	0.82
Gidlow 2008	1	2	2	2	n/a	n/a	n/a	2	2	2	1	1	2	2	0.86
Goodman 2011	1	2	2	2	n/a	n/a	n/a	2	2	2	1	2	2	2	0.91
Grasten 2015	2	2	2	1	n/a	n/a	n/a	2	2	2	1	2	2	2	0.91
Grasten 2015b	2	2	2	1	n/a	n/a	n/a	2	2	2	1	2	2	2	0.91
Groffik 2020	2	2	2	2	n/a	n/a	n/a	2	2	2	2	2	2	2	1.00
Haapala 2016	2	2	2	2	n/a	n/a	n/a	2	2	2	1	0	2	2	0.86
Hardman 2009	1	2	2	2	n/a	n/a	n/a	2	2	2	1	2	1	1	0.82
Harrington 2018	2	2	2	2	2	2	0	2	2	2	2	1	2	2	0.89
Herman 2014	2	2	2	2	n/a	n/a	n/a	2	2	2	2	2	2	2	1.00
Jago 2014	2	2	2	2	n/a	n/a	n/a	2	2	2	2	2	2	2	1.00

Jago 2017	2	2	2	2	n/a	n/a	n/a	2	2	2	2	2	2	2	1.00
Kek 2019	2	2	1	2	n/a	n/a	n/a	1	2	2	1	2	2	2	0.86
Kidokoro 2019	2	2	2	2	n/a	n/a	n/a	2	2	2	1	1	2	2	0.91
Kippe 2018	2	2	2	1	n/a	n/a	n/a	2	2	2	1	2	2	2	0.91
Kobel 2017	2	2	2	2	n/a	n/a	n/a	2	2	2	1	1	2	2	0.91
Koorts 2019	2	2	2	2	n/a	n/a	n/a	2	2	2	1	2	1	2	0.91
Long 2013	2	2	2	2	n/a	n/a	n/a	2	2	2	2	2	2	1	0.95
Loucaides 2009	1	2	1	1	1	0	0	1	2	2	1	0	1	1	0.50
Magnusson 2011	2	2	1	2	1	0	0	2	2	2	2	2	2	2	0.79
Mallam 2003	1	2	1	1	n/a	n/a	n/a	1	2	1	1	1	1	1	0.59
Mayorga-Vega 2018	1	2	2	2	n/a	n/a	n/a	2	2	2	1	2	2	2	0.91
Meyer 2013	2	2	2	2	n/a	n/a	n/a	2	2	2	2	2	2	2	1.00
Møller 2014	2	1	2	2	n/a	n/a	n/a	2	2	2	2	2	2	2	0.95
Mooses 2019	1	2	2	2	n/a	n/a	n/a	2	2	2	2	2	2	2	0.95
Morgan 2007	2	2	2	1	n/a	n/a	n/a	1	2	2	1	1	2	2	0.82
Nielsen 2012	2	2	2	2	n/a	n/a	n/a	2	2	2	2	2	2	2	1.00
O'Dwyer 2013	1	2	2	2	2	0	0	2	2	2	2	2	2	1	0.79
O'Neill 2016	2	2	1	2	n/a	n/a	n/a	2	2	2	1	1	1	1	0.77
Owen 2003	2	2	1	1	n/a	n/a	n/a	2	2	2	2	1	2	2	0.86
Palmer 2019	2	2	2	1	2	2	0	2	2	2	1	2	2	2	0.86
Pate 2016	1	2	2	2	1	0	0	2	2	2	2	2	1	2	0.75
Pelklova 2010	2	2	2	2	n/a	n/a	n/a	2	2	2	2	1	2	2	0.95
Pesola 2018	2	2	2	2	n/a	n/a	n/a	2	1	2	1	1	2	2	0.86
Ribeyre 2000	2	2	2	2	n/a	n/a	n/a	2	2	2	1	2	2	1	0.91

Ridgers 2014	2	2	1	2	n/a	n/a	n/a	2	2	2	2	1	2	2	0.91
Ridgers 2015	2	2	2	2	n/a	n/a	n/a	2	2	2	2	1	2	2	0.95
Ridgers 2017	1	2	2	2	n/a	n/a	n/a	2	2	2	2	2	1	2	0.91
Ridgers 2017b	1	2	2	1	n/a	n/a	n/a	2	2	2	2	2	2	2	0.91
Rooney 2018	2	2	1	1	n/a	n/a	n/a	2	1	2	1	1	2	2	0.77
Schneller 2017	1	2	2	1	n/a	n/a	n/a	2	2	2	1	1	2	2	0.82
Schneller 2017b	2	2	2	2	n/a	n/a	n/a	2	2	2	1	1	2	2	0.91
Sigmund 2014	2	2	2	2	n/a	n/a	n/a	2	2	2	1	1	2	2	0.91
Smith 2012	2	2	2	2	n/a	n/a	n/a	2	2	2	2	2	2	2	1.00
Stewart 2017	1	2	2	2	n/a	n/a	n/a	2	2	2	1	1	2	2	0.86
Stylianou 2016	2	2	2	1	n/a	n/a	n/a	2	2	2	1	2	2	2	0.91
Sutherland 2017	2	2	2	1	2	2	0	2	2	2	2	1	2	2	0.86
Toftager 2014	2	2	2	2	2	0	0	1	2	2	2	2	2	2	0.82
Trapp 2013	1	2	1	1	n/a	n/a	n/a	2	2	2	2	1	1	1	0.73
Tudor-Locke 2006	2	2	2	2	n/a	n/a	n/a	1	2	2	1	1	1	1	0.77
Vale 2011	2	2	2	2	n/a	n/a	n/a	2	2	2	1	1	1	2	0.86
Van Cauwenberghe 2013	2	2	2	1	n/a	n/a	n/a	2	2	2	1	2	1	1	0.82
Wilson 2017	2	2	1	1	n/a	n/a	n/a	2	2	2	1	1	2	2	0.82
															Mean: 0.86
															SD: 0.10

Frequencies														
**2**	55	76	63	52	7	4	0	69	70	75	36	44	61	62
**1**	22	1	14	25	9	1	1	8	7	2	41	31	16	15
**0**	0	0	0	0	0	11	15	0	0	0	0	2	0	0
**n/a**	0	0	0	0	61	61	61	0	0	0	0	0	0	0

Abbreviation List

n/a not applicable
